# The Chinese Version of the Patient Education Materials Assessment Tool for Printable Materials: Translation, Adaptation, and Validation Study

**DOI:** 10.2196/39808

**Published:** 2023-05-18

**Authors:** Yi Shan, Meng Ji, Zhaogang Dong, Zhaoquan Xing, Ding Wang, Xiangting Cao

**Affiliations:** 1 School of Foreign Studies, Nantong University Nantong China; 2 School of Languages and Cultures, The University of Sydney Sydney Australia; 3 Department of Clinical Laboratory, Qilu Hospital of Shandong University Ji'nan China; 4 Department of Urology, Qilu Hospital of Shandong University Ji'nan China

**Keywords:** actionability, adaptation, Chinese version of the PEMAT-P, comprehensibility, health education materials translation, validation

## Abstract

**Background:**

Providing people with understandable and actionable health information can considerably promote healthy behaviors and outcomes. To this end, some valid and reliable scales assessing the patient-friendliness of health education materials, like the PEMAT-P (Patient Education Materials Assessment Tool for printable materials), have been well developed in English-speaking countries. However, the English version of the PEMAT-P has not been translated and adapted into simplified Chinese and validated in mainland China.

**Objective:**

This study sought to translate the PEMAT-P tool into a simplified Chinese (Mandarin) version (C-PEMAT-P, a Chinese version of the Patient Education Materials Assessment Tool for printable materials) and verify its validity and reliability for assessing the comprehensibility and actionability of health education resources written in simplified Chinese. As a result, the validated C-PEMAT-P could be used to guide health researchers and educators to design more comprehensible and actionable materials for more tailored and targeted health education and interventions.

**Methods:**

We translated the PEMAT-P into simplified Chinese in the following three steps: (1) forward-translating the PEMAT-P into simplified Chinese, (2) back-translating the simplified Chinese version into English, and (3) testing translation equivalence linguistically and culturally by examining the original English version of the PEMAT-P and the back-translated English version of the tool. Any discrepancies between the original English tool and the back-translated English tool were resolved through a panel discussion among the research team of all authors to produce a revised forward-translated Chinese version (C-PEMAT-P). We then evaluated the clarity of construction and wording as well as the content relevance of the C-PEMAT-P using a 4-point ordinal scale to determine its content validity. After that, 2 native Chinese speakers (health educators) used the C-PEMAT-P to rate 15 health education handouts concerning air pollution and health to validate their reliability. We calculated the Cohen coefficient and Cronbach α to determine the interrater agreement and internal consistency of the C-PEMAT-P, respectively.

**Results:**

We finalized the translated Chinese tool after discussing the differences between the 2 English versions (original and back-translated) of the PEMAT-P, producing the final Chinese version of the PEMAT-P (C-PEMAT-P). The content validity index of the C-PEMAT-P version was 0.969, the Cohen coefficient for the interrater scoring agreement was 0.928, and the Cronbach α for internal consistency was .897. These values indicated the high validity and reliability of the C-PEMAT-P.

**Conclusions:**

The C-PEMAT-P has been proven valid and reliable. It is the first Chinese scale for assessing the comprehensibility and actionability of Chinese health education materials. It can be used as an assessment tool to evaluate health education materials currently available and a guide to help health researchers and educators design more comprehensible and actionable materials for more tailored and targeted health education and interventions.

## Introduction

### Background

Developing accurate, comprehensible, and actionable health information is essential for delivering quality, safe health care [1]. Such information can help patients know their conditions, communicate with health providers and make decisions on medical actions. However, health education materials remain too complex to comprehend for many people, although policy makers and health care providers pay growing attention to them [2]. These materials are usually poorly comprehended by patients, particularly those who have inadequate health literacy [3,4] to “obtain, process, and understand basic health information and services needed to make appropriate health decisions” [5]. Many adults lack the skills necessary to engage in their health care successfully, making health literacy a well-recognized challenge for public health [2]. Inadequate health literacy is closely related to limited disease control, medical adherence, and health outcomes [3,6]. Regardless of health literacy levels, health education materials can be effective only when the intended readers are capable of reading, understanding, and accepting the provided information [7]. Patient-friendly health education materials are, therefore, essential for patients to take medical actions and improve health outcomes.

Patient-friendly health education materials must be, first and foremost, readable. Readability means the ease by which readers are capable of reading and comprehending text [8]. This concept has long been integrated into the development and assessment of educational materials [9]. Readability formulas widely adopted to assess the reading difficulty of education materials overlook other factors influencing people’s ability to understand the information provided [10]. Education materials developers need to go beyond the only standard of readability levels [11] when determining the appropriateness of patient education materials for different readers [12,13].

Recognizing the weakness of readability formulas, researchers developed some checklists and instruments that can evaluate the health literacy demand of education materials [13-17]. However, none of these tools measure the actionability of health education materials [2], which is an essential characteristic of education materials required in the *National Action Plan* [1].

Developed to address this shortcoming of the existing instruments, the PEMAT (Patient Education Materials Assessment Tool), which has been proven valid and reliable, was designed to enable laypeople and health professionals to evaluate the comprehensibility and actionability of printable and audiovisual health education materials [2,9]. “Patient education materials are understandable when consumers of diverse backgrounds and varying levels of health literacy can process and explain key messages” [2]. “Patient education materials are actionable when consumers of diverse backgrounds and varying levels of health literacy can identify what they can do based on the information presented” [2]. Accordingly, the PEMAT has 2 versions: the PEMAT-P for printable materials (brochures and PDFs) and the PEMAT-A/V for audiovisual materials (videos and multimedia materials, including smartphone apps). Both scales comprise a scoring sheet and a user’s guide.

The PEMAT-P assesses both comprehensibility and actionability. This scale comprises 24 items: 17 related to comprehensibility and 7 related to actionability. Comprehensibility is evaluated in terms of content, word choice and style, the use of numbers, organization, layout, and design, and the use of visual aids [2]. Actionability evaluates whether the material is actionable, that is, whether the material can effectively guide patients to take due medical actions. Each of the 24 items is rated in light of the PEMAT-P evaluation criteria in which 1 point is set for “Agree,” 0 points is set for “Disagree,” and N/A is set for items that do not apply to the material. To score a material, a rater needs to (1) calculate the total scores for the material on the understandability items only or the actionability items only, (2) divide that total by the number of items on which the material has been rated, excluding the items scored “Not Applicable,” and (3) multiply the result by 100 [2]. In this way, 2 scores can be provided for the comprehensibility and actionability of each material. A high score means a high degree of comprehensibility or actionability of the material assessed. The cutoff value for understandability and actionability is set at 70% by the developers.

The PEMAT was widely applied to studies in English- and non–English-speaking countries (see Multimedia Appendix 1), enabling health care practitioners to select or develop suitable education materials for readers with varying health literacy [18-25]. Some studies reported the interrater reliability of one or both domains [18-21], the interrater reliability of the items classified by topic [22], or the interrater reliability at the item level of the PEMAT-P [23]. Some studies used the PEMAT to identify problems in patient education materials, for example, by evaluating web-based education materials for patients who took nonvitamin K oral anticoagulants [24]. Some studies used the PEMAT to develop or improve patient education materials, for example, to develop “an integrated diabetes-periodontitis nutrition and health education module” [25].

Although well-developed assessment tools are adopted for evaluating the suitability of health education materials in English-speaking countries, such instruments are hardly applied in Chinese-speaking communities [7]. Without a ready-made assessment tool, translating existing scales into different languages is a rapid and practical approach [7]. The original English version of the PEMAT-P has been translated into Malay [26], Korean [27], and Japanese [28]. However, a simplified Chinese (Mandarin) version has not been developed through translation to assess the comprehensibility and actionability of Chinese health education materials.

### Objective

This study sought to translate and adapt the PEMAT-P into a Mandarin Chinese version (C-PEMAT-P, a Chinese version of the Patient Education Materials Assessment Tool for printable materials) and verify its validity and reliability for evaluating whether Chinese health education materials are understandable and actionable. In this first stage of development, we intended to test the validity and reliability of the C-PEMAT-P among health educators, whose professional background in public health education and practical experience in engaging with patients could facilitate the development and adaptation of the C-PEMAT-P and ensure the quality of the tool we developed. In the next stage of development, we will conduct further research to test both the PEMAT and the Chinese version with a reasonably sized sample of end users on some target materials, to establish the equivalence of scores.

## Methods

### Translation of the PEMAT-P

**Figure 1:**
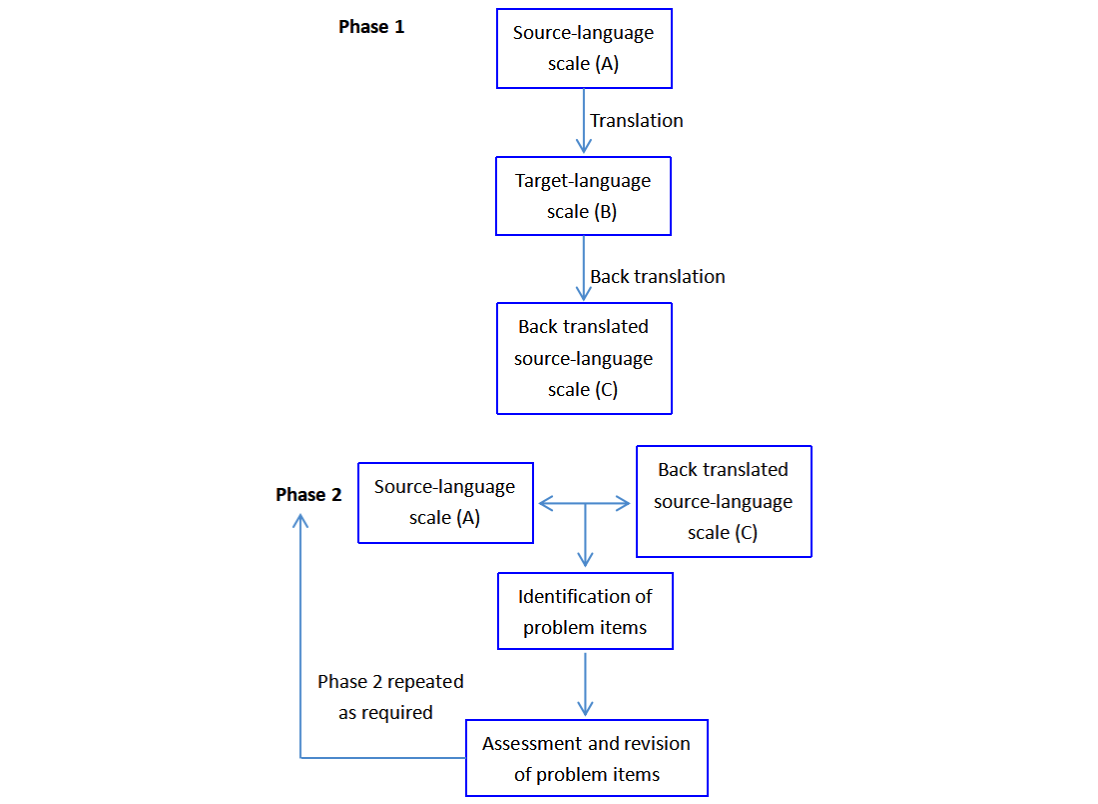
The whole process of developing the simplified Chinese version of DISCERN.

Previous studies adopted forward-translation, back-translation, bilingual testing, and monolingual testing in the scale translation process, which are essential for studies involving cross-cultural comparisons [29]. Informed by these techniques, we translated the PEMAT-P into Mandarin Chinese to ensure semantic, pragmatic, and cultural equivalence following the rigorous procedures used in the model proposed by Sperber et al [30], as shown in Figure 1.

#### Forward-Translation

The PEMAT-P scoring sheet and user’s guide were translated from English into Chinese by a native Chinese speaker. An experienced bilingual translator was then requested to review and identify problematic words, phrases, or sentences in the translated Chinese version independently. Afterward, we discussed the reviewer’s comments and suggestions with the translator and revised the Chinese version. In this process, we paid close attention to cultural appropriateness, which means the correspondence of the core concepts in the materials with the logic, language, and experiences of the target culture and the use of positive cultural images and examples [12].

#### Back-Translation

To minimize errors in back-translation, we selected a translator carefully [31]. An experienced bilingual translator translated the revised Chinese version into English. This back-translator was blinded to the original English tool and different from the forward translator.

#### Translation Equivalence Testing

Inspired by Sperber et al’s [30] translation equivalence testing approach to validating translated instruments, we validated the revised initially translated Chinese tool by comparing the 2 English versions (original- and back-translated) in the comparability of language (CL) and the similarity of interpretability (SI). “Comparability of language refers to the formal similarity of words, phrases, and sentences. The similarity of interpretability refers to the degree to which the two versions would engender the same response even if the words are not the same” [30]. A native English speaker, currently teaching at a university in China, compared the 2 English versions in CL and SI to identify problematic items. He was asked to rank CL and SI, using an ordinal scale of 1=extremely comparable, 2=comparable, 3=not comparable, and 4=not at all comparable for CL, and an ordinal scale of 1=extremely similar, 2=similar, 3=not similar, and 4=not at all similar for SI [7], respectively. A panel comprising 2 bilingual health educators familiar with the PEMAT-P and the authors discussed and corrected problematic items in the Chinese version rated as 3 or 4. All problematic items that had been corrected were then retranslated until they were comparable and interpreted in the same way in both languages.

#### Final Chinese Version

Based on cultural appropriateness [12], we then consulted an experienced bilingual translator to finalize the Chinese version.

### Psychometric Properties Testing

Content validation sought to assess the item relevance in each domain and the clarity of the translated items [32]. During the validation, a panel of experts gave constructive feedback on the quality of the newly developed scale and objective criteria for assessing each item involved [33].

Four health educators (ZD, ZX, DW, and XC) participated in the psychometric properties testing of the C-PEMAT-P. They all have a background in public health education. ZX and ZD were highly qualified health educators who have been working as professors and doctors at Qilu Hospital of Shandong University, China because they received their doctorates at Shandong University. DW and XC are currently studying for their master’s degree in public health education at Shandong University. Their professional educational background and experience in engaging with patients at Qilu Hospital of Shandong University can qualify them for the content validation and reliability testing of the newly developed tool.

### Content Validity

Four Chinese health educators (ZD, ZX, DW, and XC) were requested to assess the content validity of the finalized Chinese version (C-PEMAT-P) by evaluating the clarity and relevance of the items in the C-PEMAT-P. We calculated the clarity of construction and wording as well as the content relevance using a 4-point ordinal scale of 1=item is not clear, 2=item needs major revisions to be clear, 3=item needs minor revisions to be clear, and 4=item is clear [7] for clarity, and a 4-point ordinal scale of 1=item is not relevant, 2=item needs major revisions to be relevant, 3=item needs minor revisions to be relevant, and 4=item is relevant for relevance. We summed the items rated as 3 or 4 by the 4 health educators and divided the sum by the total number of items rated to measure the content validity index [33].

### Reliability Testing

#### Interrater Reliability

The interrater reliability was tested to determine the scoring consistency between raters. We invited 2 health educators (ZD and ZX) to assess the comprehensibility and actionability of 15 print health education materials on air pollution and health using the C-PEMAT-P. Cohen coefficient was calculated to evaluate the interrater agreement.

#### Internal Consistency

Four health educators (ZD, ZX, DW, and XC) were requested to verify the internal consistency of the C-PEMAT-P using a selected print material on air pollution and health. We calculated Cronbach α to assess the internal consistency of the C-PEMAT-P.

### Data Collection and Analysis

Print scoring sheets and rating sheets were used to collect data manually. Quantitative analyses were conducted using SPSS (version 22.0; IBM Corp) to determine the content validity index, Cohen coefficient for interrater reliability, and Cronbach α for internal consistency.

### Ethical Considerations

This study was approved by the ethics review board of Qilu Hospital of Shandong University, China (KYLL-202208-026).

## Results

### Comparison of the Two English Versions

According to Sperber et al’s [30] translation validation method, the native English speaker compared the 2 English versions (original and back-translated) in respect of CL and SI. Table 1 shows the results of the comparison.

**Table 1:** Comparison of the original and back-translated English versions of the Patient Education Materials Assessment Tool for printable materials in terms of comparability of language and similarity of interpretability. The scores in bold indicate problematic items.

	Original English version	Back-translated English version	Comparability of language	Similarity of interpretability
1	The material makes its purpose completely evident.	The article has an apparent purpose.	2	1
2	The material does not include information or content that distracts from its purpose.	The article does not contain irrelevant content.	2	1
3	The material uses common, everyday language.	The article uses everyday language.	1	1
4	Specialized terms are used only to familiarize audience with the terms. When used, specialized terms are defined.	Specialized terms are used only to familiarize the reader with the words. If specialized terms are used, the author also gives explanations and definitions.	1	1
5	The material uses the active voice.	This article uses the active voice.	1	1
6	Numbers appearing in the material are clear and easy to understand.	The numbers in the article are clear and easy to understand.	1	1
7	The material does not expect the user to perform calculations.	Readers do not need to do the calculations themselves.	2	1
8	The material breaks or “chunks” information into short sections.	This article breaks down large paragraphs of information into smaller paragraphs of content.	**3**	2
9	The material’s sections have informative headers.	The title of the article makes it easy to see what the article is about.	**4**	2
10	The material presents information in a logical sequence.	The content of the article is logical.	**3**	1
11	The material provides a summary.	The article has a summary.	1	1
12	The material uses visual cues (eg, arrows, boxes, bullets, bold, larger font, and highlighting) to draw attention to key points.	The article uses visual graphics (eg, arrows, etc.) to emphasize the main points of the article.	2	2
13	The material uses visual aids whenever they could make content more easily understood (eg, illustration of healthy portion size).	The article often uses diagrams (eg, a healthy diet nutrition chart) to help people understand the content more easily.	2	1
14	The material’s visual aids reinforce rather than distract from the content.	The diagrams emphasize the main content rather than distract the reader.	1	1
15	The material’s visual aids have clear titles or captions.	The diagrams have clear headings or notes.	2	2
16	The material uses illustrations and photographs that are clear and uncluttered.	The pictures and photos are clear and easy to recognize.	2	1
17	The material uses simple tables with short and clear row and column headings.	The diagrams are simple, with short and clear row and column headings.	1	1
18	The material clearly identifies at least one action the user can take.	The article clearly presents at least one action that the reader can take.	2	1
19	The material addresses the user directly when describing actions.	When the article recommends action, it clearly points out who the target audience is.	**3**	1
20	The material breaks down any action into manageable, explicit steps.	When recommending actions, the article further decomposes the actions into clear, actionable, concrete steps.	**3**	2
21	The material provides a tangible tool (eg, menu planners, checklists) whenever it could help the user take action.	The article provides simple, easy-to-plan, record-keeping tools (eg, recipe planners, checklists) for readers to take action.	2	1
22	The material provides simple instructions or examples of how to perform calculations.	The article gives simple examples of how to do the calculation.	1	1
23	The material explains how to use the charts, graphs, tables, or diagrams to take actions.	The article explains how to use various diagrams to take action.	1	1
24	The material uses visual aids whenever they could make it easier to act on the instructions.	The article uses diagrams as much as possible to make it easier for readers to follow the suggested actions.	2	1

Nineteen of the 24 items were rated as *extremely similar*, and the remaining 5 were rated as *similar*. Therefore, all 24 items achieved an acceptable degree of SI and did not need to be corrected in this aspect. Concerning CL, 19 of the 24 items were rated either as *extremely comparable* or as *comparable*, but 4 (items 8, 10, 19, and 20) were rated as *not comparable*, and 1 (item 9) was rated as *not at all comparable*. To make these 5 items in the back-translated version more comparable with the corresponding items in the original version, we revised these items in the Chinese version and back-translated them into English as follows:

8. This article breaks down the whole passage into smaller sections.

9. The section headings of this article make it easy to see what the article is about.

10. The content of the article is presented logically.

19. The article directly tells the reader the recommended actions.

20. The article breaks down the recommended actions into clear, actionable clear steps.

Considering cultural appropriateness [12], we avoided the literal translation of “chunks,” “information,” “header,” “address,” and “informative” in the revision of the Chinese version. We asked the same native English speaker to rate the 5 back-translated English items above. He rated all of them as *comparable*. As a result, all 24 items passed the translation equivalence testing.

### Psychometric Properties Testing

#### Content Validity

Four Chinese health educators evaluated the content validity of the C-PEMAT-P. For the whole C-PEMAT-P, the content validity index in clarity was 0.969, and the content validity index in relevance was also 0.969. Of the 24 items on the C-PEMAT-P scoring sheet, 3 items (4, 6, and 24) received a 0.75 rating for item clarity, and the remaining items received a 1.0 rating for item clarity. Three items (4, 6, and 24) received a 0.75 rating for content relevance, and the others received a 1.0 rating for content relevance. One of the 4 health educators gave items 4, 6, and 24 a score of 2, 1, and 2, respectively, for clarity. The same health educator gave a score of 2 to each of these 3 items for relevance. We did not further revise these items for 2 reasons: (1) the other 3 health educators all gave these 3 items a score of 4 for clarity and relevance, and (2) we concluded that these 3 items were not problematic after checking them against the original English version of the PEMAT-P.

#### Reliability Testing

##### Interrater Reliability

Two raters assessed 15 printed health education materials on air pollution and health independently using the C-PEMAT-P. The Cohen coefficient for the interrater agreement was .944 (P=.0<.05). For the 15 materials rated, 19 (79%) of the 24 items in the C-PEMAT-P were given the same score by the 2 raters. Five (21%) of the 24 items (6, 7, 10, 12, and 21) received different scores in 8 materials out of those 15 materials assessed.

##### Internal Consistency

The Cronbach α was determined at .897 for the internal consistency of the overall Chinese scale. For comprehensibility, Cronbach α was determined at .847. For actionability, Cronbach α was determined at .751.

## Discussion

### Principal Findings

#### Translation

This study followed the cross-cultural translation model (comprising forward-translation, back-translation, and translation equivalence testing) that was proposed by Sperber et al [30] for health instrument translation. We found that the model was effective in the translation of the PEMAT-P into the C-PEMAT-P. This finding confirms the findings reported by some recent studies [34-37], which also attested to the effectiveness of this model in the cross-lingual, cross-cultural translation of health-related instruments and materials. We adopted back-translation to verify the translation equivalence of the C-PEMAT-P by requesting a native English speaker to compare the back-translated English version and the original English version of the PEMAT-P in terms of CL and SI, therefore, minimizing translation errors. The errors we identified and corrected in the translation stage confirmed the translation errors proposed by Capitulo et al [38], which were elaborated in the following subsection of Comparison with Previous Studies. Most of these errors stemmed from some cultural aspects that may not be captured through linguistic translation alone. Therefore, we propose some essential cultural aspects that need to be considered in the cross-cultural translation of health scales, as listed and glossed in Table 2.

**Table 2:** Essential cultural aspects to be considered in the cross-cultural translation of health scales.

Cultural aspects	Gloss
Cultural equivalence	Functional equivalence between the source and target scales that is achieved through well considering the types of the original text, the importance of cultural color in the original text, the purpose of translation, and the reader type of the target text.
Cultural appropriateness	Correspondence of the core concepts in the materials with the logic, language, and experiences of the target culture and the use of positive cultural images and examples.
Similarity of interpretability	The degree to which the source-language scale and the target-language scale would engender the same response even if the words are not the same.
Item relevance	Relevance of the items of the translated scale to the corresponding subscales (if any) and the intended domain of the translated scale.

#### Validity

Through the content validity testing of the C-PEMAT-P, we obtained a high content validity index of 0.969, which verified the validity of this newly developed instrument for evaluating the comprehensibility and actionability of Chinese health education materials on air pollution and health.

#### Reliability

Cohen coefficient of .994 obtained for the interrater reliability implied a fair scoring agreement between the 2 raters. This interrater reliability was relatively higher than that in previous studies [9,18-23,28]. Analyzing the rating scores for the 24 items provided by the 2 raters, we found that high interrater agreement was associated with less subjective rating criteria. The reliability test of the C-PEMAT-P identified inconsistent scoring between the 2 raters on items 6, 7, 10, 12, and 21. These findings revealed that we could refine and reassess the subjective scoring criteria for the C-PEMAT-P that generated relatively lower interrater agreement [7].

### Comparison With Previous Studies

Various methods were used to translate assessment tools in previous studies [29]. Regardless of the methods adopted, finding qualified translators is the first step in the translation process [7]. It is usually difficult to find competent bilingual translators familiar with the semantic content of the scale [31]. Translators are not always adequately knowledgeable in the subject area of a scale, and some even translate the original text literally without paying due attention to cultural nuances [7]. In this respect, colloquial phrases, slang, and jargon terms challenge translators the most [39]. In this study, the translator of the Chinese instrument is a competent bilingual of Chinese and English who has engaged in medical informatics and translation in this field for several years. Taking cultural nuances and appropriateness into account, she translated the original English version of the PEMAT-P into Chinese, achieving a high degree of semantic, pragmatic, and cultural equivalence.

Back-translation is a helpful approach to detecting translation errors [31,40]. These errors included (1) the addition of words or phrases to the original, (2) the deletion of words or phrases from the original, (3) the alteration of the original meaning by changing words or phrases, and (4) the use of poor grammar and syntax impacting meaning and clarity negatively [39]. In the PEMAT-P, some technical terms, for example, “active voice,” “chunks,” “specialized terms,” “visual aids,” etc, were challenging to understand when translated literally into Chinese. Instead, the translator conveyed their meanings using culturally equivalent words, phrases, or sentences, which, however, possibly risked distorting the original intention of the source text [39]. Informed by Yu et al [40], we adopted back-translation, which allowed for comparing the 2 English PEMAT-P versions (original- and back-translated), to minimize the risk of translation distortion. As a result, we identified all potential problematic items, ensuring a finalized Chinese version of optimal translation equivalence, the C-PEMAT-P.

A few previous studies that used the PEMAT to assess the comprehensibility and actionability of health education materials described interrater reliability. Studies using 2 or more raters usually calculated Cohen coefficient [9,18-23,28]. Cohen coefficients of <0.2, 0.21-0.4, 0.41-0.6, 0.61-0.8, and more than 0.8 are regarded as poor, fair, moderate, strong, and nearly complete agreement, respectively [41]. Because experience in assessing print health education materials can affect interrater reliability [42], we invited health educator raters experienced in assessing print health education materials, contributing to relatively higher interrater reliability than previous studies [9,18-23,28]. Considering that “there is latitude allowed in the interpretation of the criteria” that may cause subjectivity in rating materials [43], we propose that training programs should be carried out to increase the raters’ experience in evaluating health education materials to raise the interrater reliability for the C-PEMAT-P.

### Limitations

This study has some limitations. First, although we went through rigorous procedures to translate, adapt, and validate the C-PEMAT-P, probably, we could not eliminate all potential linguistic and cultural discrepancies. Second, we used the C-PEMAT-P to assess the comprehensibility and actionability of health education materials from the health educators’ perspective, but we failed to verify these 2 aspects from the patients’ perspective. Therefore, future studies need to invite non–health care professionals as informants who will use the C-PEMAT-P to assess the comprehensibility and actionability of health education materials. The third limitation is directly associated with the second limitation. Future research needs to be conducted to measure the actual behavioral change and the resulting health outcomes by following up with patient informants for a given period after the intervention. Fourth, we used the newly developed Chinese tool to assess health education materials only on air pollution and health. Further studies need to be conducted to evaluate other health education materials to verify the usability of the new tool. Finally, concurrent validity was not assessed due to the lack of validated Chinese-language scales similar to the PEMAT-P. However, we adopted the back-translation strategy, the translation equivalence testing, the content validity testing, and the reliability testing to ensure the validity and reliability of the C-PEMAT-P.

### Conclusions

To address the unavailability of patient education materials assessment tools in Chinese-speaking communities, we translated and cross-culturally adapted the PEMAT-P into the C-PEMAT-P and verified its reliability and validity for evaluating the comprehensibility and actionability of health education materials written in Chinese. The C-PEMAT-P is the first validated Chinese-language scale for assessing the comprehensibility and actionability of Chinese health education materials. It has the potential to allow health care educators and practitioners to provide patients and the public with Chinese health education materials that are easy to understand and act on. It can also enable them to improve or develop patient-friendly health education materials. As a result, patients and the public cannot only increase their perceived self-efficacy but also promote their actual behavioral change to achieve desired health outcomes. In the future, it is imperative to verify the generalizability of the findings of this study and further validate the C-PEMAT-P by conducting many similar studies.

## Appendix

Multimedia Appendix 1 The Chinese version of the PEMAT-P (the original English version of the PEMAT-P can be consulted at [44]).

## Abbreviations

CL: comparability of language
C-PEMAT-P: a Chinese version of the Patient Education Materials Assessment Tool for printable materials
PEMAT-P: the Patient Education Materials Assessment Tool for printable materials
SI: similarity of interpretability

## Data Availability

Data are available upon reasonable request via victorsyhz@hotmail.com.
